# Response to comment on: Evaluating the impact of nitisinone at mosquito-lethal doses on *Lutzomyia longipalpis*

**DOI:** 10.1371/journal.pntd.0014381

**Published:** 2026-07-02

**Authors:** Laure Augendre, Lucas Alexandre Farias de Souza, Magda Clara Vieira da Costa Ribeiro, Jérôme Depaquit, Jean-Philippe Martinet, Jorian Prudhomme

**Affiliations:** 1 UR ESCAPE Epidemiosurveillance and circulation of parasites in the environment, University of Reims Champagne-Ardenne, Faculty of Pharmacy, Reims, France; 2 Insects Vectors and Parasites Laboratory, Department of Basic Pathology and Postgraduate program in Microbiology, Parasitology and Pathology, Federal University of Paraná, Curitiba, Brazil; 3 Laboratoire de Parasitologie-Mycologie, Centre Hospitalo-Universitaire, pôle de Biologie territoriale, Reims, France; London School of Hygiene and Tropical Medicine, UNITED KINGDOM OF GREAT BRITAIN AND NORTHERN IRELAND

We thank the authors of this commentary for their careful reading of our work and for their thoughtful remarks. We fully agree that inhibitors of the tyrosine degradation pathway, such as nitisinone, represent promising candidates for innovative vector management strategies across a range of hematophagous arthropods. We welcome the opportunity to clarify aspects of our experimental approach and the interpretation of our findings.

First, regarding the choice of nitisinone concentration, our objective was not to establish a comprehensive dose–response relationship in *Lutzomyia longipalpis*. As stated in our article, our study was designed as an exploratory evaluation to determine whether a concentration previously reported to be lethal in mosquitoes could also induce mortality in the sand fly species *Lu. Longipalpis*, the main vector of *Leishmania infantum* in the Americas. At the time of the study, no sand fly specific susceptibility data were available, and the selected concentration was therefore intended as a biologically informed starting point rather than a definitive estimate of lethality. Establishing species-specific dose–response relationships will be the focus of future studies following this initial validation.

We acknowledge that including a higher, clearly lethal concentration as a positive control could have strengthened interpretability. However, the primary aim of our study was to test whether a mosquito-relevant concentration could produce measurable effects in sand flies under standard laboratory conditions. Our findings should therefore be interpreted as evidence that, under the tested conditions, this concentration did not induce significant mortality in *Lu. longipalpis*, rather than as a definitive demonstration of intrinsic tolerance to nitisinone.

The commentary suggests that the absence of mortality observed in our experiments is most likely attributable to insufficient dosing. While this represents a plausible explanation, it is not the only interpretation. The assertion that the tested concentration is “well below experimentally established lethal thresholds” is largely based on extrapolations across distantly related taxa with markedly different physiology and feeding biology. While cross-species comparisons can provide useful context, they cannot replace direct empirical observations. Our results indicate that the concentration derived from mosquito studies did not produce measurable mortality in *Lu. longipalpis* under the conditions used.

Comparisons across hematophagous arthropods should also be interpreted cautiously. Differences in physiology, feeding behavior, metabolism, and detoxification pathways can strongly influence susceptibility to xenobiotics. Consequently, lethal thresholds established in mosquitoes or tsetse flies cannot be directly extrapolated to sand flies. Accordingly, our study tested the concentration derived from mosquito studies empirically, rather than assuming equivalence across species.

The commentary also raises important points regarding differences in blood-meal volume between mosquitoes and sand flies. As noted in our article, we agree that the total ingested dose is influenced by feeding volume and digestion physiology, which may vary substantially among hematophagous insects. Our intention in referencing mosquito data was not to assume direct equivalence between taxa but rather to provide a comparative framework in the absence of prior sand fly studies. And consequently, our experimental design therefore focused on testing whether the nominal concentration used in mosquito studies would produce detectable effects in sand flies. We agree that future investigations would benefit from quantitative measurements of blood-meal volume and dose scaling specifically adapted to sand fly feeding biology. This will constitute the next step following the initial validation reported in our publication.

Regarding feeding conditions, starvation duration, and feeding success, we acknowledge that additional detail on feeding rates and blood-meal ingestion would improve interpretability. In our experiments, the feeding rate in the untreated control group was 64% in replicate A and 39% in replicate B. In the nitisinone-treated group, feeding rates were 54% in replicate A and 59% in replicate B. Sand flies were maintained under standard colony conditions commonly used for *Lu. longipalpis* laboratory rearing, including sugar starvation only 24h prior to blood feeding. These conditions reflect routine experimental practice in sand fly biology. While such factors may influence feeding dynamics, they do not invalidate the observed survival outcomes.

We also agree that direct confirmation of nitisinone ingestion or downstream metabolic effects would provide additional mechanistic insight. Our study focused on assessing organism-level survival outcomes following exposure through the blood meal. Direct biochemical confirmation of ingestion or metabolic disruption was therefore beyond the scope of this exploratory investigation. Future studies incorporating metabolomic or physiological analyses could provide important insights into the pharmacodynamics of nitisinone in sand flies.

Finally, the experiments were conducted using independent biological replicates consistent with exploratory studies in sand fly systems. As it is well recognized in the scientific community, sand flies are more challenging to maintain than mosquitoes, which inherently limits the feasibility of obtaining large sample sizes in this experimental model. While larger datasets would undoubtedly increase statistical power, the purpose of this initial experiment was to determine whether a detectable mortality signal could be observed at the tested concentration. For this exploratory study, the sample size was appropriate to generate meaningful preliminary data, and the absence of a detectable signal under these conditions can inform the design of future dose–response experiments.

In summary, as stated in our article, our study was intended as an initial exploratory evaluation of whether a nitisinone concentration previously shown to be lethal to mosquitoes could induce mortality in *Lutzomyia longipalpis*. The results indicate that, under the tested conditions, this concentration did not produce significant mortality in sand flies. We agree with the commentators that further work—including species-specific dose–response experiments, quantitative assessment of blood-meal ingestion, and confirmation of internal exposure—will be necessary to fully evaluate the susceptibility of sand flies to HPPD inhibitors. While additional studies are needed to determine species-specific dose–response relationships and investigate pharmacological mechanisms, the empirical observations reported here remain valid within the defined scope of the study.

